# Recent insights of obesity-induced gut and adipose tissue dysbiosis in type 2 diabetes

**DOI:** 10.3389/fmolb.2023.1224982

**Published:** 2023-09-28

**Authors:** Debarun Patra, Dipanjan Banerjee, Palla Ramprasad, Soumyajit Roy, Durba Pal, Suman Dasgupta

**Affiliations:** ^1^ Department of Biomedical Engineering, Indian Institute of Technology Ropar, Punjab, Punjab, India; ^2^ Department of Molecular Biology and Biotechnology, Tezpur University, Napaam, Assam, India

**Keywords:** adipose tissue, dysbiosis, gut microbiota, insulin resistance, obesity, type 2 diabetes

## Abstract

An imbalance in microbial homeostasis, referred to as dysbiosis, is critically associated with the progression of obesity-induced metabolic disorders including type 2 diabetes (T2D). Alteration in gut microbial diversity and the abundance of pathogenic bacteria disrupt metabolic homeostasis and potentiate chronic inflammation, due to intestinal leakage or release of a diverse range of microbial metabolites. The obesity-associated shifts in gut microbial diversity worsen the triglyceride and cholesterol level that regulates adipogenesis, lipolysis, and fatty acid oxidation. Moreover, an intricate interaction of the gut-brain axis coupled with the altered microbiome profile and microbiome-derived metabolites disrupt bidirectional communication for instigating insulin resistance. Furthermore, a distinct microbial community within visceral adipose tissue is associated with its dysfunction in obese T2D individuals. The specific bacterial signature was found in the mesenteric adipose tissue of T2D patients. Recently, it has been shown that in Crohn’s disease, the gut-derived bacterium *Clostridium innocuum* translocated to the mesenteric adipose tissue and modulates its function by inducing M2 macrophage polarization, increasing adipogenesis, and promoting microbial surveillance. Considering these facts, modulation of microbiota in the gut and adipose tissue could serve as one of the contemporary approaches to manage T2D by using prebiotics, probiotics, or faecal microbial transplantation. Altogether, this review consolidates the current knowledge on gut and adipose tissue dysbiosis and its role in the development and progression of obesity-induced T2D. It emphasizes the significance of the gut microbiota and its metabolites as well as the alteration of adipose tissue microbiome profile for promoting adipose tissue dysfunction, and identifying novel therapeutic strategies, providing valuable insights and directions for future research and potential clinical interventions.

## 1 Introduction

Obesity is a severe human health problem that increasing globally at an alarming rate which significantly contributes to the development of several metabolic diseases including type 2 diabetes (T2D) (Pulgaron and Delamater, 2014). According to the World Health Organization report, more than 2 billion adults worldwide are overweight or obese, and the number of people with diabetes has risen from 151 million in 2000 to 537 million in 2021 (Ruze et al., 2023). Recent evidence suggests that diet and food habits strikingly influence the pathophysiological state of obesity potentiating the onset and progression of T2D (Guo et al., 2020).

The gut microbiota iscomprisedof about 10 trillion different bacteria that increase in density as these moves along the gastrointestinal (GI) tract (Cani et al., 2019). The host and gut microbiota represent a symbiotic connection in which the host provides a nutrient-rich habitat for the microbiome community while the microbes influence the host’s physiology, immunology, and metabolism (Thursby and Juge, 2017). The dynamic complexity of the microbial ecosystem and its composition is influenced by several factors, including diet and lifestyle, which play a vital role in the maintenance of host health by regulating the immune system, metabolizing nutrients, and producing essential vitamins and hormones (Hou et al., 2022). Moreover, the gut microbiome critically regulates the gut-brain axis wherein gut microbial metabolites such as short-chain fatty acids (SCFAs) and neurotransmitters can cross the blood-brain barrier and influence brain function (Silva et al., 2020).

Gut dysbiosis, a condition characterized by an imbalance in the composition and function of the gut microbiome, has been implicated in several chronic diseases, including inflammatory bowel disease, colorectal cancer, and T2D (Vijay and Valdes, 2022). Understanding the relationship between the gut microbiome and insulin resistance has been improved in the last few decades. Gut dysbiosis and the disrupted gut permeability (leaky gut) in obesity allow lipopolysaccharide (LPS) and other pro-inflammatory molecules to enter the bloodstream and trigger systemic inflammation, contributing to the progression of insulin resistance and T2D (Boulangé et al., 2016). Obesity has been attributed to several metabolic changes that are causally linked with glucose intolerance and insulin resistance associated with T2D (Martyn et al., 2008; Wondmkun, 2020). Few recent studies revealed the enrichment of microorganisms in mesenteric and omental adipose tissue during obesity, which acts as a key driver of adipose tissue inflammation that potentiates Crohn’s disease and insulin resistance (Ha et al., 2020; Massier et al., 2020). Thus, targeting the gut microbiome has emerged as a promising strategy for the prevention and treatment of obesity-induced T2D. Several therapeutic strategies, such as probiotics, prebiotics, and dietary modifications, have been proposed to modulate the gut microbiome to prevent or treat T2D (Huda et al., 2021).

This review aims to provide an overview of the current understanding of gut and adipose tissue dysbiosis in obesity-induced T2D and to identify future research directions and associated challenges in this field. We explored the mechanisms by which adipose tissue microbiome enriched in obesity and associated gut dysbiosis that correlated with altered gut microbiota-brain axis, and pancreas dysfunction through the emergence of microbial metabolites contributing to the development of T2D. We also examined the shreds of evidence supporting the use of various therapeutic strategies aimed at modulating the gut microbiome.

## 2 Gut dysbiosis linked with obesity and type 2 diabetes

The human gut accommodates a diverse community of enteric microflora, including Firmicutes and Bacteroidetes (up to 75% of total gut flora), which is also called a “virtual organ”, provides structural, metabolic, and protective benefits to intestinal epithelial cells (O’Hara and Shanahan, 2006; Foster and McVey Neufeld, 2013). The human gut environment can be viewed as a dynamic system formed by the host and its microbiota working together. The gut microbiota involves in carbohydrate and fat metabolism, vitamin and amino acid production, the proliferation of epithelial cells, defense against infections, and hormone regulation in the host body (Oliphant and Allen-Vercoe, 2019) and also helps in the digestion of plant polysaccharides, complex nutrients, and milk sugars (Xu and Gordon, 2003; Hersoug et al., 2018) those which were not properly digested by the host and thus contributing 10% of caloric value and aid in preserving intestinal health and anti-cancer properties (Wong et al., 2006; Blustein et al., 2013; Cox and Blaser, 2013).

### 2.1 Obesity induces alteration of gut microbiota profile

Obesity is a complex metabolic disorder resulting from an imbalance between energy intake and energy expenditure (Hill et al., 2012). It is associated with dysregulation of lipid and glucose metabolism leading to abnormal levels of blood lipids causally linked with hyperlipidemia, ectopic lipid deposition, glucose intolerance, insulin resistance, and T2D (Klop et al., 2013). Reportedly, lipid has bidirectional regulation with the gut microbiota. Dietary lipids break down into fatty acids that are majorly absorbed in the GI tract and are found to modulate bacterial diversity as several fatty acids exhibit antibiotic activity and reduce ATP production (Jackman et al., 2016; Schoeler and Caesar, 2019). In the distal colon, residual peptides and proteins, bile acids, and choline undergo fermentation that produces a more diverse range of products compared to the fermentation of carbohydrates in the proximal colon (Canfora et al., 2022). Some of the products generated in the distal colon include bacterial toxins such as LPS, hydrogen sulfide, bile acid derivatives like deoxycholate and lithocholate, branched-chain amino acids (BCAAs) and their metabolites, and branched-chain fatty acids (BCFAs) like isobutyrate, 2-methyl butyrate, and isovalerate (Zhai et al., 2021). Additionally, aromatic amino acids (AAAs) give rise to phenolic, indolic, skatolic, and p-cresolic compounds, ammonia, and polyamines. Choline, another substrate, produces dimethylamine (DMA) and trimethylamine (TMA) as end products in the distal colon (Zhai et al., 2021) (Table 1).

**TABLE 1:** Microbial metabolites and their role in obesity and T2D pathogenesis.

Metabolites	Source microorganism	Status in obesity and T2D	Role in pathogenesis	Ref
Styrylpyrone-type metabolite penstyrylpyrone	*Penicillium* sp.	Decrease	Improves disease condition	Lee et al. (2013); Mar Rodríguez et al. (2015)
Butyrate	*Fusobacterium, Eubacterium biforme, Butyrivibrio crossotus, Clostridium symbiosum, Roseburia, Anaerostipes, Coprococcus, Faecalibacterium*	Decrease	improves colon mucosal barrier function, exhibits immunomodulatory effects and anti-inflammatory properties by downregulating pro-Inflammatory cytokines	Segain et al. (2000); Canfora et al. (2019); Hermes et al. (2020); Kim et al. (2020); Palmas et al. (2021)
BCAAs (Branched-chain amino acid) (valine, leucine, isoleucine)	*Fusobacterium*	Increase	Insulin resistance	Newgard et al. (2009); Reddy et al. (2018); Kim et al. (2020)
Endotoxin	*Lactobacillus* spp.	Decrease	Improvement of mucosal barrier function	Diamant et al. (2011)
Linoleic acid	*Bifidobacteria*	Decrease	Increase omega-3 fatty acid levels in Adipose tissue and reduce the pro-inflammatory cytokines	Diamant et al. (2011)
SFCA	*Ruminococcus gnavus, Eubacterium biforme, Butyrivibrio crossotus, Clostridium symbiosum, Roseburia, Anaerostipes, Coprococcus, Faecalibacterium, Ruminococcus, Phascolarctobacterium, Dialister, Megasphaera*	Decrease	Suppress weight gain, Glucose-stimulated insulin secretion, increases GLP-1 and peptide YY (PYY)	Lin et al. (2012); Vallianou et al. (2018); Canfora et al. (2019); Hermes et al. (2020); Palmas et al. (2021)
LPS	*Enterobacter, Escherichia albertii*	Increase	Metabolic endotoxemia, inflammation	Cani et al. (2007); de la Cuesta-Zuluaga et al. (2018); Palmas et al. (2021)
Hydrogen sulfide	*Desulfovibrio piger*	Increase	Pro-inflammatory effects and Toxic intestinal epithelial cells	Palmas et al. (2021)
Polyamines	*Clostridium, Peptostreptococcus, Peptococcus*	Increase	Inflammation	Canfora et al. (2019); Bui et al. (2022)
BCFAs	*Bacteroides, Eubacterium, Clostridium*	Increase	Inflammation and dyslipidemia	Canfora et al. (2019)

Specific gut bacteria, such as *Akkermansia muciniphila* and *Bifidobacterium*, have been shown to produce metabolites that directly affect glucose metabolism leading to increased insulin sensitivity and improved glucose tolerance. On the contrary, obesity-associated changes in gut bacteria have been shown to produce metabolites that can promote insulin resistance and glucose intolerance. Mice feeding with an HFD comprising 49.5% lipid content reduce *Bifidobacterium* spp., *Eubacterium rectale*, *Clostridium coccoides* as well as *Bacteroides* (Cani et al., 2007). Both high-fat and high-sugar diet feeding significantly altered the gut microbiota diversity, promoting the accumulation of Gram-negative bacteria (Cani et al., 2007). About 50% reduction in *Bacteroidetes* and a proportional increase in *Firmicutes* have been identified in genetically obese mice models (Ley et al., 2005).

Many bacterial genera are either positively or negatively correlated with obese T2D conditions. The overabundance of bacterial genera including *Fusobacterium*, *Ruminococcus*, and *Blautia* was found to be positively correlated with the pathophysiology of T2D (Table 2). In contrast, several bacterial genera including *Faecalibacterium*, *Bifidobacterium*, *Akkermansia*, *Roseburia*, and *Bacteroides* were negatively correlated with T2D and therefore beneficial to counteract the pathogenesis of T2D (Gurung et al., 2020) (Table 2). Certain gut bacterial populations were linked to metabolic alterations, body fat mass, and calorie consumption. The gut bacteria *Faecalibacterium prausnitzii* was reported to be linked with shifts in inflammatory status and insulin sensitivity (Furet et al., 2010). *F. prausnitzii* is relatively attuned to urinary metabolites, corpulence, and inflammation, and exhibits anti-inflammatory effects by targeting the NF-κB pathway. This bacteria species is recognized as a preserved and prominent species of healthy individuals’ faecal microbiota (Li et al., 2008; Sokol et al., 2008; Tap et al., 2009; Furet et al., 2010). *Lactobacillus rhamnosus* CNCM I-3690 as a probiotic candidate counteracts *B. Wadsworth*-mediated immunological dysfunction and also supports bolstering the intestinal barrier and lowering inflammation (Natividad et al., 2018). *Blautia wexlerae* administration reduces obesity and T2D through metabolic gut microbiota reconstruction (Hosomi et al., 2022). The microbial community regulates hormonal balance, bowel permeability, expression of genes regulating lipogenesis, insulin resistance, endotoxemia, interaction with bile acids, and changes in the distribution of brown adipose tissue (BAT) (Cornejo-Pareja et al., 2019). Moreover, an unbalanced gut flora in obese people might cause chronic inflammation followed by metabolic diseases including insulin resistance and T2D (Singh et al., 2017). People even trying to improve gut barrier function by using herbal medicine like Bofutsushosan causally associated with the blooms *Akkermansia muciniphila* and improves glucose metabolism in mice (Fujisaka et al., 2020). Nuciferine, a main bioactive component in the lotus leaf, when treated with HFD rats showed a significant decline in the ratio of Firmicutes/Bacteroidetes phyla which resulted in reduced blood endotoxemia and inflammation along with increased intestinal integrity and SCFA synthesis (Wang et al., 2020). Interestingly, blood glucose levels may also be adversely affected by the oral microbial compositions that cause both local and systemic inflammation (Omori et al., 2022). Alteration in the salivary microbiome has also been reported in T2D patients with a higher abundance of *Streptococcus* sp., *Lactobacillus* sp., *Blautia wexlerae*, *Lactobacillus fermentum*, *Nocardia coeliaca*, *Selenomonas artemidis* (Kampoo et al., 2014; Long et al., 2017) and reduction of phylum Actinobacteria, and Bifidobacterium (Kampoo et al., 2014; Long et al., 2017).

**TABLE 2:** Summary of microbial signature at different taxonomic level in the gut and adipose tissue of obesity and type 2 diabetes.

Genus	Species	Obesity & type 2 diabetes	Adipose tissue	Gut	References
Faecalibacterium	prausnitzii	Increase	-	Yes	Furet et al. (2010)
Akkermansia	muciniphila	Decrease	-	Yes	Roopchand et al. (2015)
Akkermansia	-	Increase	-	Yes	Parks et al. (2013)
*Clostridium*	*clostridioforme*	Increase	-	Yes	Karlsson et al. (2013); Parks et al. (2013)
Bifidobacterium	-	Decrease	-	Yes	Furet et al. (2010)
*Mycobacterium*	tuberculosis	Increase	Yes		Neyrolles et al. (2006)
Lactoococcus	-	Increase	-	Yes	Peroumal et al. (2022)
Allobaculum	-	Increase	-	Yes	Peroumal et al. (2022)
*Allistipes*	*-*	Decrease	-	Yes	Verdam et al. (2013)
*Bacteroides*	*ovatus*	Decrease	-	Yes	Verdam et al. (2013)
*Oscillospira*	*guillermondii*	Decrease	-	Yes	Verdam et al. (2013); Konikoff and Gophna (2016); Gophna et al. (2017); Palmas et al. (2021)
*Desulfovibrio*	*piger*	Increase	-	Yes	Konikoff and Gophna (2016)
*Barnesiella*	-	Decrease	-	Yes	Parks et al. (2013)
*Eubacterium*	*hallii*	Decrease	-	Yes	Verdam et al. (2013)
*Lactobacillus*	*plantarum*	Increase	-	Yes	Van Baarlen et al. (2009); Diamant et al. (2011); Sedighi et al. (2017)
*Dorea*	*formicigenerans*	Increase	-	Yes	Verdam et al. (2013)
*Bacteroides*	*plebeius*	Decrease	-	Yes	Verdam et al. (2013)
*Bacteroides*	*splachnicus*	Decrease	-	Yes	Verdam et al. (2013)
*Parabacteroides*	*distasonis*	Decrease	-	Yes	Verdam et al. (2013)
*Clostridium*	*symbiosum*	Increase	-	Yes	Verdam et al. (2013)
*Escherichia*	*albertii*	Increase	-	Yes	Palmas et al. (2021)
*Bacteroides*	*caccae*	Increase	-	Yes	Qin et al. (2012)
*Clostridium*	*hathewayi*	Increase	-	Yes	Qin et al. (2012)
*Clostridium*	*ramosum*	Increase	-	Yes	Qin et al. (2012)
*Clostridium*	*symbiosum*	Increase	-	Yes	Qin et al. (2012)
*Eggerthella*	*lenta*	Increase	-	Yes	Qin et al. (2012)
*Escherichia*	*coli*	Increase	-	Yes	Qin et al. (2012)
*Granulicatella*	*-*	Increase	-	Yes	Aranaz et al. (2021)
*Veillonella*	*-*	Increase	-	Yes	Aranaz et al. (2021)
*Haemophilus*	*-*	Increase	-	Yes	Aranaz et al. (2021)
*Dialister*	*-*	Increase	-	Yes	Aranaz et al. (2021)
*Parabacteroides*	*-*	Increase	-	Yes	Aranaz et al. (2021)
*Prevotella*	*-*	Increase	-	Yes	Aranaz et al. (2021)
*Shigella*	*-*	Increase	-	Yes	Aranaz et al. (2021)
*Allisonella*	*-*	Increase	-	Yes	Aranaz et al. (2021)
*Blautia*	*-*	Increase	-	Yes	Zeng et al. (2019)
*Romboutsia*	*-*	Increase	-	Yes	Zeng et al. (2019)
*Ruminococcus*	*-*	Increase	-	Yes	Zeng et al. (2019)
*Clostridium*	*sensu stricto*	Increase	-	Yes	Zeng et al. (2019)
*Oscillibacter*	*valericigenes*	Increase	Yes	-	Li et al. (2022)
*Trypanosoma*	*cruzi*	Increase	Yes	-	Tanowitz et al. (2017)
*Trypanosoma*	*brucei*	Increase	Yes	-	Tanowitz et al. (2017)
*Plasmodium* spp.	*-*	Increase	Yes	-	Tanowitz et al. (2017)
*Ottowia*	*-*	Increase	Yes	-	Massier et al. (2020)
*Xanthomonas*	*-*	Increase	Yes	-	Massier et al. (2020)
*Nosocomiicoccus*	-	Increase	Yes	-	Massier et al. (2020)
*Paenibacillus*	-	Increase	Yes	-	Massier et al. (2020)
*Rhodotorula*	*-*	Increase	-	Yes	Mar Rodríguez et al. (2015)
*Aspergillus*	*-*	Increase	-	Yes	Mar Rodríguez et al. (2015)
*Atopobium*	*-*	Increase	Yes	-	Massier et al. (2020)
*Allorhizobium*	*-*	Increase	Yes	-	Massier et al. (2020)
*Delftia*	*-*	Increase	Yes	-	Massier et al. (2020)
*Bdellovibrio*	*-*	Increase	Yes	-	Massier et al. (2020)
*Acinetobacter*	*-*	Increase	Yes	-	Massier et al. (2020)
*Alicycliphilus*	*-*	Increase	Yes	-	Massier et al. (2020)
*Jeotgalicoccus*	*-*	Increase	Yes	-	Massier et al. (2020)
*Exiguobacterium*	*-*	Increase	Yes	-	Massier et al. (2020)
*Gemella*	*-*	Increase	Yes	-	Massier et al. (2020)
*Candida*	*metapsilosis*	Increase	Yes	-	Massier et al. (2020)
*Malassezia*	*restricta*	Increase	Yes	-	Massier et al. (2020)
*Pseudozyma*	*aphidis*	Increase	Yes	-	Massier et al. (2020)
*Candida*	albicans	Increase	-	Yes	Peroumal et al. (2022)
*Eurotium*	*-*	Increase	-	Yes	Mar Rodríguez et al. (2015)
*Human adenovirus-36*	*-*	-	Yes	-	Dhurandhar et al. (1997); Atkinson et al. (2005)

### 2.2 Gut microbial-derived metabolites modulate lipid metabolism in obesity

Microbiota-derived metabolites, such as bile acids, LPS, and SCFAs have been identified as important factors in the regulation of hyperlipidemia (Agus et al., 2021; Jia et al., 2021). SCFAs such as acetic acid, propionic acid, butyric acid, isobutyric acid, valeric acid, and isovaleric acid are produced from the fermentation of dietary fibre by gut microbes and are known to be associated with several health benefits (Rios-Covian et al., 2020). These fatty acids also influence adipogenesis, lipolysis, and fatty acid oxidation, all of which are related to lipid metabolism in non-obese and obese states (Breton et al., 2022). Propionate has been found to increase the expression of peroxisome proliferator-activated receptors (PPARs), which are key regulators of adipogenesis (Hong et al., 2005). Gut microbiota is known to inhibit adenosine monophosphate kinase (AMPK) activity, an enzyme that plays a crucial role in energy homeostasis, leading to the reduction of fatty acid oxidation coincided with higher cholesterol and triglycerides favouring lipogenesis. High-fat diet (HFD)-fed germ-free (GF) mice showed higher levels of phosphorylated AMPK and promoted fatty acid oxidation, compared to control animals (Bäckhed et al., 2007). Microbiota transfer from conventional (CV) mice to GF mice has been found to affect the fasting-induced adipose factor, angiopoietin-like 4 (ANGPTL4). ANGPTL4 inhibits lipoprotein lipase (LPL), which decreases the accumulation of triglycerides in adipocytes and relevantly regulates fat storage (Bäckhed et al., 2004).

**FIGURE 1:**
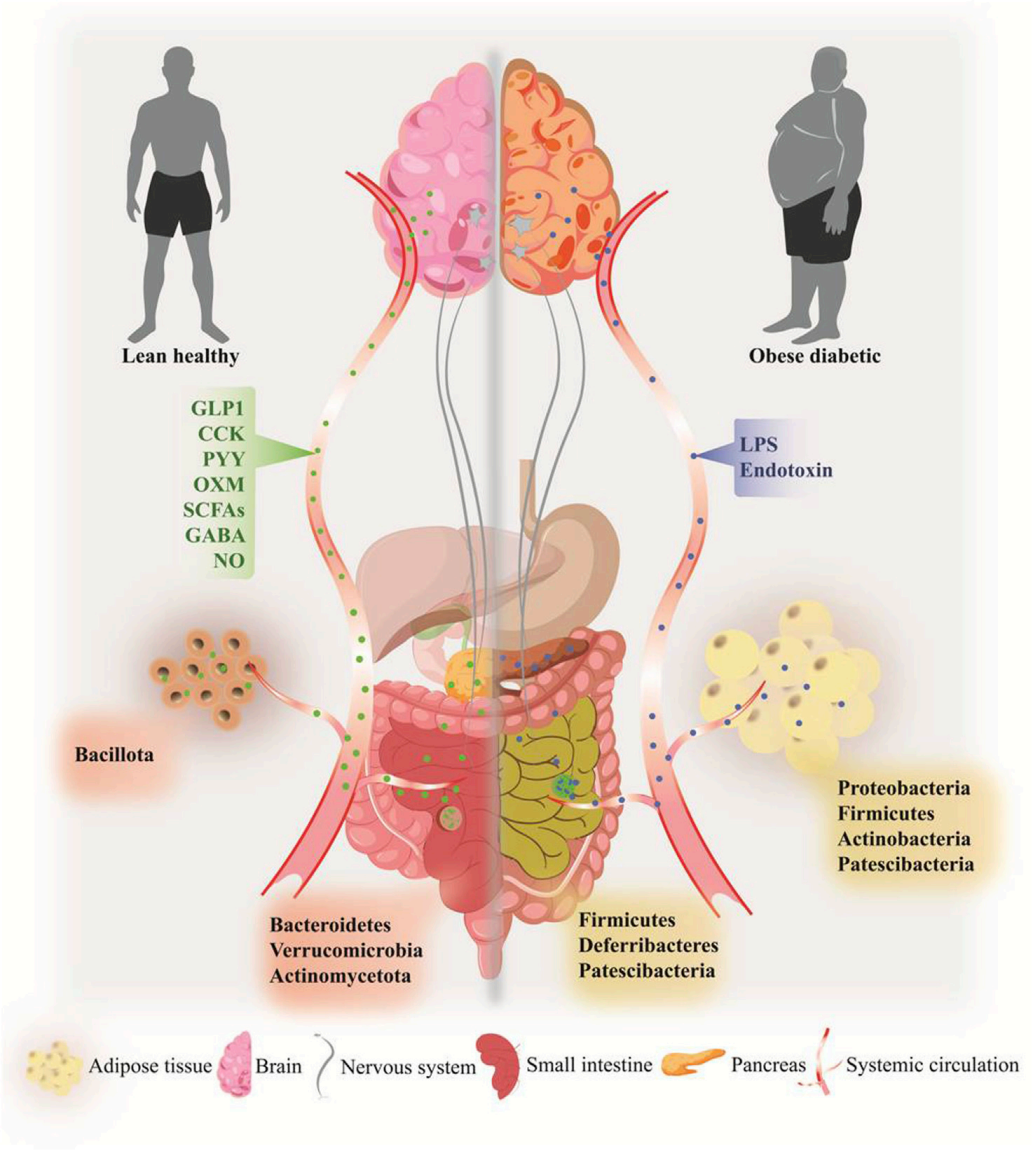
Overview of obesity-induced dysbiosis and associated pathogenesis in the gut, gut-brain axis, pancreas, and adipose tissue of type 2 diabetes. Specific microbial phyla signatures were observed in gut and adipose tissue in the onset of obesity-induced T2D (right side) compared to lean healthy individual (left side). A Plethora of gut microbiome-derived metabolites participates in organs function, including gut-brain homeostasis, pancreas function, and inflammatory homeostasis. Alteration of microbial metabolites including LPS, endotoxins, and MAMPs leads to the development of pathophysiological state in obesity and T2D, by rapid systemic inflammation, releasing pro-inflammatory molecules, gut-brain dysfunction, and pancreatic damages.

Obesity-associated gut microbial imbalance leads to alterations in the production of these metabolites and signalling molecules causing T2D pathogenesis (Figure 1). It has been shown that a specific gut microbiota community belonging to the Prevotella group produced a small metabolite succinate that improved glucose homeostasis in mice by regulating intestinal gluconeogenesis (Ghanim et al., 2009). Similarly, it has been found that a membrane protein from *A. muciniphila*, a mucin-degrading gut bacterium, protected mice from obesity and associated complications (Buckley et al., 2014). Moreover, a novel microbially produced small molecule imidazole propionate is involved in the impairment of insulin signalling by targeting the mammalian target of rapamycin complex 1 (mTORC1) and thus promoting glucose intolerance and insulin resistance (Kindt et al., 2010). Furthermore, alteration of microbiota profile as well microbial leakage causes pancreatic infection and inflammation that impairs the production of insulin and promotes insulin resistance leading to glucose intolerance and T2D. Transplantation of faecal matter from Western diet-fed or genetically obese mice was capable of generating an obese phenotype in the standard diet-fed or non-obese mice, which resulted in a larger weight gain compared to treatment with wild-type microorganisms (De Groot et al., 2020). Furthermore, an obese pathophysiological state decreases the number of *A. muciniphila* by increasing NAD and riboflavin-biosynthesis. Together, these functional adjustments particularly allow glutathione to be recharged to its reduced state, enabling redox balance in microorganisms that may be exposed to the gut which is a potentially unfriendly, inflammatory, and oxidatively challenged environment (Yassour et al., 2016). It has also been observed that Bacteroidetes were decreased in number with a concomitant increase in Firmicutes in the gut of HFD-fed mice. This alteration in the bacterial balance may have a role in the emergence and development of obesity (Magne et al., 2020). Firmicutes bacteria are believed to have a greater capacity to obtain energy from food, which could increase caloric intake. On the contrary, Bacteroidetes are linked with weight management either by oxidation of SCFAs or the digestion of complex carbohydrates (Singh et al., 2017). Moreover, by transferring faecal microbiota from hyperlipidemic individuals to GF mice, researchers have been able to observe the pathogenic effects of the microbiota on lipid metabolism (Burz et al., 2021). Velagapudi et al.compared GF mice with CV mice and found differences in triglyceride (TG) levels in various tissues. The GF mice showed lower levels of TG in adipose tissue and the liver but higher levels of TG in the circulatory system compared to CV mice (Velagapudi et al., 2010). Interestingly, western diet-fed GF mice were found to have less hypercholesterolemia (high levels of cholesterol in the blood) and increased cholesterol excretion in the liver and faeces compared to CV mice (Velagapudi et al., 2010). This indicates that the absence of gut microbiota influences cholesterol metabolism and may lead to reduced cholesterol accumulation in the blood. These studies highlight the relationship between gut microbial compositions and metabolic disorders such as T2D in HFD feeding.

### 2.3 Gut microbiome components promote chronic inflammation in obesity

**FIGURE 2:**
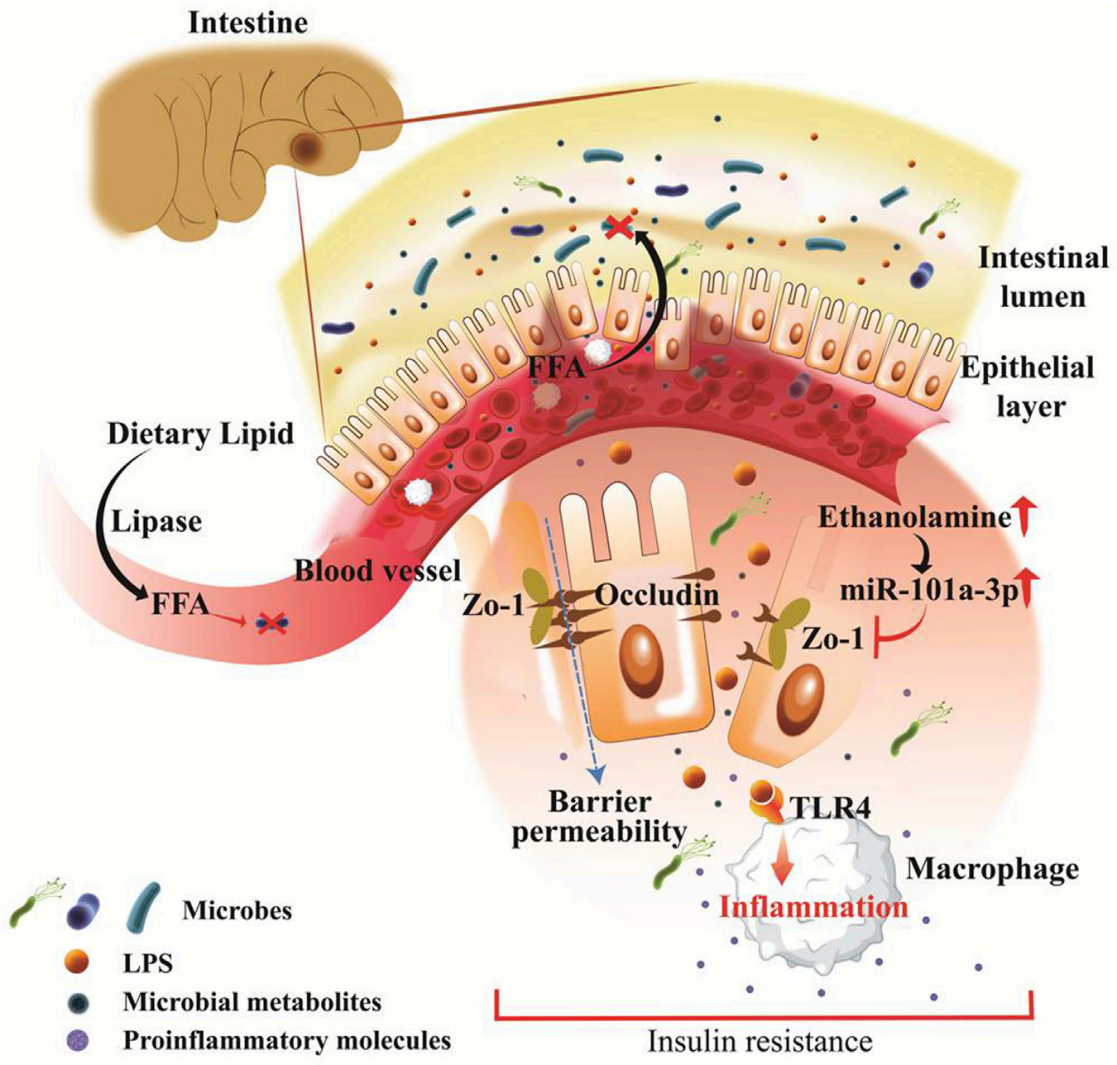
Pathophysiologically leaky gut associated with obesity and type 2 diabetes. Fat-enriched diet consumption is aligned with obesity and hyperlipidemia wherein dietary lipid breaks down into free fatty acids and absorbed in the intestine and embraces antibiotic response, and facilitates microbiome alteration in obesity. Intestinal epithelial layer barricades between gut microbiota and systemic circulation, that rapidly disrupted during gut pathogenesis. Pathogenic microbes and derived metabolites including LPS and ethanolamine are rapidly produced in obesity and directly found routes to systemic circulation. LPS endorses rapid inflammation through TLR4, CD14, and MD-2 which breaks down epithelial tight junctions. Ethanolamine activated microRNA species such as miR-101a-3p and inhibits the critical tight junction molecules zona occludens-1 (ZO-1). This result in increased barrier permeability and affiliates rapid inflammation, insulin resistance, and abnormal glucose metabolism.

Obesity is associated with chronic low-grade systemic inflammation, often regulated by microbial signature in the host, which is associated with insulin resistance, a hallmark of T2D. Inflammatory molecules produced by gut bacteria can enter the bloodstream, promoting chronic inflammation and impairing insulin signalling. LPS, a common structural component of the outer membrane of Gram-negative bacteria, reportedly promoted the onset of obesity (Boulangé et al., 2016). LPS and other bacterial debris pass from the gut environment and enter into the circulatory system due to increased intestinal permeability triggering an immune reaction. As seen by enhanced adipose macrophage recruitment and hepatic NF-κB/IKKβ inflammatory-signaling pathways, this was linked to systemic inflammation, most likely via LPS or saturated fatty acids (Takeda et al., 2003; Kleinridders et al., 2009) Mice lacking toll-like receptor 4 (TLR4) and cluster of differentiation 14 (CD14), receptors for LPS, are resistant to HFD- or LPS-induced hyperinsulinemia and insulin resistance (Stavropoulou et al., 2020). Elevated LPS content in the gut decreases the tight junction proteinzona occludens-1 (ZO-1) and occludin expression and affects the permeability and integrity of intestinal epithelial cells (Jia et al., 2021). Overall, the release of inflammatory metabolites ultimately impairs lipid metabolism. In a recent study by Mishra et al., it has been shown that gut microbiota in obese mice and humans have reduced ability for ethanolamine metabolism that resulted in increased intestinal permeability (Mishra et al., 2023). Moreover, ethanolamine elevated the abundance of a specific microRNA, miR-101a-3p, and enhanced miR promoter binding ARID3a transcription factor, which leads to the loss of a critical tight junction protein ZO-1. As a result, the weakening of the intestinal barrier leads to increased gut permeability, inflammation, and abnormalities in glucose metabolism (Figure 2). Importantly, restoration of ethanolamine-metabolizing activity can be done by correcting ARID3a/miR-101a-3p/ZO-1 axis and improving the integrity of the intestinal barrier (Mishra et al., 2023).

### 2.4 Obesity-induced gut dysbiosis influences T2D-associated pathogenesis

Gut dysbiosis is a phenomenon that refers to a disruption in the composition and function of the microorganisms that inhabit the human body. Recent studies have demonstrated that gut dysbiosis is associated with obesity and T2D, two major health concerns that affect millions of people worldwide. Obesity and T2D are closely linked, with obesity being one of the major risk factors for developing T2D. Studies have indicated that alterations in gut dysbiosis may contribute to the development of insulin resistance, which are key driver of T2D. In a landmark study, researchers transplanted gut microbiota from lean or obese human donors into GF mice. Mice receiving microbiota from obese donors showed increased total body fat and insulin resistance compared to those receiving microbiota from lean donors. This study provided early evidence for a potential link between gut microbiota and obesity-related metabolic dysfunction (Turnbaugh et al., 2006). It has been reported that the gut microbiota of obese individuals is highly efficient at extracting energy from the diet, leading to increased energy storage, which promotes the hypertrophy of adipose tissue and weight gain. The gut microbiome is structured by diet composition (David et al., 2014; Von Schwartzenberg et al., 2021), and polyunsaturated fatty acids (PUFA)-enriched dietary lipids exhibited a positive effect on the gut microbiota, restoring obesity-induced gut microbial dysfunction in mice (Haneishi et al., 2023). Multiple population studies have consistently demonstrated associations between gut microbiota composition and obesity-related metabolic disorders, including T2D (Table 3). Qin et al. found significant differences in the gut microbial composition between obese and non-obese individuals, with lower bacterial diversity observed in the gut microbiota of obese individuals (Qin et al., 2012). Specific microbial taxa associated with obesity were identified, indicating a potential role of the gut microbiota in energy harvest and metabolic dysfunction. Le Chatelier et al. also identified compositional differences in the gut microbiota between lean and obese individuals, with certain bacterial groups associated with adiposity, insulin resistance, and metabolic parameters (Le Chatelier et al., 2013). Moreover, Karlsson et al. highlighted the importance of gut microbial diversity, as reduced bacterial richness was associated with a higher prevalence of insulin resistance and metabolic disorders (Karlsson et al., 2013). A large-scale population study was performed to examine the association between gut microbiota and metabolic health, specifically focusing on obesity and insulin resistance. It identified specific gut microbial signatures, or patterns, that were associated with obesity and insulin resistance, as well as observed distinct differences in the gut microbiota composition between individuals with normal and impaired glucose metabolism which altogether highlighted the potential importance of the microbiome in the development and progression of obesity-related diabetes (Forslund et al., 2015). Several reports on animal studies also provide compelling evidence that alterations in the gut microbiota can influence metabolic health, including obesity-related diabetes. They demonstrated that manipulating the gut microbiota composition or transferring microbiota from obese individuals can lead to metabolic abnormalities in healthy animals (Cani et al., 2008; Ridaura et al., 2013; Everard et al., 2014). These studies collectively support the notion that alterations in the gut microbiota composition are associated with obesity-related diabetes and provide insight into potential mechanisms underlying this relationship (Table 3).

**TABLE 3:** Summary of predominant microbial taxa and their functions in different health conditions, such as obesity, T2D, and obesity-associated T2D.

Health state	Microbial taxa	Function	References
Obese	*Proteus mirabilis*	Potential drivers of inflammation	Xu et al. (2022)
	*E. coli*	Potential drivers of inflammation	Xu et al. (2022)
	*Veillonella*	Positively correlated with CD11b, Insulin resistance and low-grade inflammation	Moreno-Indias et al. (2016); Nirmalkar et al. (2018); Aranaz et al. (2021)
	Prevotella	Positively correlated with IL6, Insulin resistance and low-grade inflammation	Moreno-Indias et al. (2016); Nirmalkar et al. (2018); Aranaz et al. (2021)
	Succinovibrio	Positively correlated with TNF alpha	Moreno-Indias et al. (2016)
	Firmicutes	Positively correlated with IL1B	Moreno-Indias et al. (2016)
	Lachnospiraceae	Low-levels of HDL-C	Del Chierico et al. (2021); Sarmiento-Andrade et al. (2022)
	*Lactobacillus*	Associated with weight gain	Nirmalkar et al. (2018); Xu et al. (2022)
	*Blautia*	Associated with weight gain	Nirmalkar et al. (2018)
	*Collinsella*	Involved with the high levels of triglycerides and cholesterol	Nirmalkar et al. (2018)
	*Succinivibrio*		Xu et al. (2022)
	*Fusobacterium*	Mounting adhesiveness to host epithelial cells, and inflammatory responses	Ahmad et al. (2020); Xu et al. (2022)
	*Ruminococcus*	Associated with EDF markers	Nirmalkar et al. (2018)
	*Bacteroides*	Associated with EDF markers	Nirmalkar et al. (2018)
T2D	*Gammaproteobacteria*	Inflammation mainly due to endotoxins	Sroka-Oleksiak et al. (2020)
	*Lactobacillus*	Positively correlated with HBA1c and HOMA-IR	Chen et al. (2019); Ejtahed et al. (2020)
	*Escherichia*	Impaired epithelial integrity, low-grade inflammation, and autoimmune responses	Ejtahed et al. (2020)
	Prevotella	Conflicting effects on glycemic control	Ejtahed et al. (2020)
	*Bacteroides ovatus*	Impaired glucose tolerance	Del Chierico et al. (2021); Sarmiento-Andrade et al. (2022)
	Enterobacteriaceae	Impaired glucose tolerance	Del Chierico et al. (2021); Sarmiento-Andrade et al. (2022)
	Fusobacteria	Mounting adhesiveness to host epithelial cells, and inflammatory responses	Ahmad et al. (2020)
	*Bacteroides vulgatus*	Increase plasma IL-6 which is linked to low-grade inflammation and insulin resistance	Leite et al. (2017)
	*Prevotellacopri*	Increase plasma IL-6 which is linked to low-grade inflammation and insulin resistance	Leite et al. (2017)
Obesity-associated T2D	Enterobacteriaceae	Bacterial load in plasma, liver, and omental adipose tissue leads to inflammation	Anhê et al. (2020); Xu et al. (2022)
	Acidobacteria	Positive association with the diabetes state	Ahmad et al. (2020)
	Deferribacteres	Positive association with the diabetes state	Ahmad et al. (2020)
	Gemmatimonadetes	Positive association with the diabetes state	Ahmad et al. (2020)
	Gammaproteobacteria	Inflammation	Ahmad et al. (2020)
	Dialister	Mediates an inflammatory response and insulin resistance	Ahmad et al. (2020)
	Allisonella	Mediates an inflammatory response and insulin resistance	Ahmad et al. (2020)
	Fusobacteria	Mounting adhesiveness to host epithelial cells, and inflammatory responses	Ahmad et al. (2020)

It is also worth mentioning that the field of gut microbiota research is evolving, and new studies may provide additional insights into the specific mechanisms and relationships between microbial compositions and metabolic disorders. These findings highlight the important role of gut microbiota composition in metabolic disorders and provide valuable insights into specific bacterial genera that may be associated with either positive or negative outcomes in terms of metabolic health.

## 3 Adipose tissue dysbiosis promotes adipocyte dysfunction in obesity-induced type 2 diabetes

Adipose tissue is a metabolically active endocrine, immunological, energy storage organ critically regulating energy balance and glucose metabolism. The enriching complexity of adipose tissue is associated with its microbiome, which consists of a diverse community of microorganisms that reside within the tissue microenvironment and appear to play an important role in regulating adipose tissue inflammation and insulin sensitivity.

### 3.1 Microbiota-derived factors regulate adipose tissue function

Adipose tissue dysbiosis can also influence adipose tissue function by altering the production and release of various adipokines. Apart from the tissue microbiome, the intestinal microbial population was found to regulate adipocyte function. Genetically obese mice (ob/ob) inherit alteration in the microbiota with increasing Firmicutes population in comparison to lean heterozygous (ob/+) animals. Moreover, the microbial composition is very similar to the obese human samples (Ley et al., 2005). The fat-storing capacity is critically regulated by the microbiome and GF mice are protected from obesity even after being fed an HFD (Bäckhed et al., 2004). The absence of a microbiome leads to an overexpression of Fasting-Induced Adipose Factor (*Fiaf*), a protein involved in lipid metabolism by inhibiting the activity of lipoprotein lipase (LPL), an enzyme responsible for breaking down triglycerides. Additionally, it induces the expression of PGC-1α and activates AMPK, both of which impact cellular energy metabolism (Bäckhed et al., 2005). These combined effects enhance adipocyte lipid storage, potentially leading to increased fat accumulation. Few microbes become therapeutically significant as of their impact on leanness; such as the administration of *Akkermansia muciniphila*, *Lactobacillus plantarum* reportedly reduces the impact of an obesogenic diet on human and animals (Depommier et al., 2019; Heeney et al., 2019). Faecalmicrobiota transplantation (FMT) experiments involving the transfer of intestinal microbiota from obese patients into GF mice have provided evidence that microbiota transfer correlates with obesity. To minimize the inter-individual variability of FMT transplantation, a recent study was performed by FMT on the same morbidly obese patient before and after undergoing (RYGB) bariatric surgery in GF mice. The results showed that RYGB surgery led to improving metabolic health along with the changes in the microbiota composition in both patients and the mice receiving the FMTs from pre- and post-surgery stools compared to mice with pre-surgery microbiota. Moreover, visceral adipose tissue (VAT) and subcutaneous white adipose tissue (scWAT) function improved by reducing the expression of *Tnf-a*, *Ccl2*, *Elane* (neutrophil elastase), and increasing anti-inflammatory Sirtuin1 level in the post-RYGB FMT mice along with improvement of BAT function by enriching UCP1 positive areas, anti-inflammatory markers IL33, IL2Ra expression (Yadav et al., 2023). Another subset of microbiome-derived products including propionate, flavonoid, tryptophan-derived metabolites, and cell wall components actively regulates adipose tissue. Tryptophan serves as an essential amino acid, playing a vital role as a building block for various proteins. However, it is worth noting that tryptophan has additional significance beyond its role in protein synthesis. The microbiota, residing in the gut, possesses the ability to convert tryptophan into indole compounds. These indole compounds can accumulate in the gut lumen. A recent study investigated the impact of tryptophan-derived metabolites on a group of miRNAs known as the miR-181 family, which are known to be upregulated in obese white adipose tissue. Interestingly, deletion of the two most highly expressed miR-181 clusters in mice protected them from developing obesity caused by an HFD. A specific tryptophan-derived metabolite called indole-3-carboxylic acid was identified as being reduced in mice fed an HFD. It was found that this metabolite acts on adipocytes to inhibit the expression of miR-181. By regulating miR-181 expression, indole-3-carboxylic acid was shown to have an impact on energy expenditure and insulin sensitivity, suggesting its involvement in the regulation of metabolism (Virtue et al., 2019). Emerging evidence suggeststoll-like receptor (TLR) ligands and nucleotide-binding oligomerization domain-containing (NOD) proteins, specifically NOD1 and NOD2, play crucial roles in adipose tissue dysfunction (Chan et al., 2017). An abundance of microbiota-derived LPS causes activation of TLR signalling in adipocytes and other immune cells, which in turn results secretion of pro-inflammatory cytokines and chemokines, promoting a state of chronic low-grade inflammation; a characteristic feature of adipose tissue dysfunction observed in conditions such as obesity and associate with insulin resistance. Moreover, NOD1 and NOD2, members of the NOD-like receptor (NLR) family, are present in both adipocytes and immune cells within adipose tissue and play significant roles in adipose tissue dysfunction. Activation of NOD1 is associated with insulin resistance, whereas NOD2 null mice develop chronic inflammation and insulin resistance without change in adiposity upon being fed an HFD (Denou et al., 2015; Chan et al., 2017; Martínez-Montoro et al., 2022). Overall, these pattern recognition receptors contribute to adipose tissue inflammation, metabolic dysregulation, and impaired insulin sensitivity.

### 3.2 Microbial compartmentalization in the white adipose tissue depots during obesity

Similar to the intestinal microbiome, recent studies revealed independent adipose tissue microbiome exists and obese people exhibit an increase in Firmicutes and a reduction in Bacteroidetes. Among the different depots of adipose tissue, mesenteric white adipose tissue (mWAT) harbours the highest bacterial quantity and diversity. The mWAT is a continuous band of adipose tissue that wraps around the various segments of the intestines and acts as a gateway for the intestines to communicate with the rest of the body’s systems. Moreover, the adipose tissue microbiome’s composition and functionality are known to vary with obesity which regulates inflammation and metabolic dysfunction (Davis, 2016; Massier et al., 2020; Zheng et al., 2020). Burcelin et al. introduced the initial hypothesis of the “tissue microbiota” after finding bacterial DNA in different metabolic organs such as the liver, and adipose tissue of human beings (Burcelin et al., 2013). Moreover, the presence of bacterial DNA in distinct compartmentalization within the mesenteric adipose tissueand omental adipose tissue is associated with type 2 diabetes (T2D) (Anhê et al., 2020). Later, the bacterial diversity was compared in the white adipose tissue (WAT) of subcutaneous, visceral, and mesenteric depots and it was found bacterial population is predominant in mesenteric WAT (Massier et al., 2020). These findings suggest that WAT may operate as a critical institution to host bacterial populations and to metabolic dysfunction under obesity-associated T2D (Table 2).

Microbiota-mediated interruptions in OXPHOS/mitochondria lead to functional impairment of white adipose tissue, the primary governing factor of systemic glucose metabolism (Li et al., 2022). Consumption of a high-fat/high-sugar diet leads to the expansion of specific bacteria that produce TLR2 ligands, triggering the induction of Mmp12+ macrophages in WAT. Mmp12+ macrophages serve as a link between microbiota-dependent inflammation and OXPHOS damage in WAT. These macrophages exhibited a specific molecular signature that was associated with insulin resistance in obese patients and released MMP12, which acts as a bridge between inflammation and mitochondrial damage in WAT, ultimately leading to insulin resistance (Li et al., 2022).

Alteration in the microbiota may have a role in both chronic inflammation and insulin resistance (Boulangé et al., 2016; Foley et al., 2018; Kawai et al., 2021). Studies have shown that specific bacterial species prevalent in the adipose tissue of obese people can create inflammatory mediators like LPS, which can cause insulin resistance and other metabolic diseases (Massier et al., 2020). LPS plays a crucial role in macrophage polarization, specifically in the transition from an anti-inflammatory to a pro-inflammatory phenotype. The exposure of adipocytes to LPS, potentially influenced by their size, may play a role in adipocyte cell death with the formation of crown-like structures in inflamed adipose tissue. Furthermore, it has been observed that LPS present within adipocytes can activate caspase-4/5/11, which can induce a highly inflammatory form of programmed cell death known as pyroptosis; typically associated with intracellular pathogen infections (Hersoug et al., 2018). Changing microflora composition in obese adipose tissue exhibits similarities to the altered gut microbiome. In overweight individuals, studies have shown that adipose tissue contains higher amounts of bacterial strains such as Proteobacteria and Actinobacteria (Massier et al., 2020). These changes in the composition of adipose tissue microbiota may have implications for metabolic health. Further analysis using 16s rRNA sequencing revealed specific bacterial families in the VAT of obese and lean individuals. The presence of Streptococcaceae and Ruminococaceae families, along with other unnamed genera, was observed in obese VAT, and Marvinobryantia and Bacilliales were found in lean VAT (Shantaram et al., 2022). It is worth noting that the presence of Streptococcaceae and Ruminococaceae in the VAT has been associated with inflammatory milieu and the recruitment of VAT-specific neutrophils, suggesting potential implications for adipose tissue inflammation. Studies have also reported the presence of bacteria like *Mycobacterium tuberculosis*, parasites such as *Trypanosoma cruzi*, *Trypanosoma brucei*, *Plasmodium berghei*, and pathogens like *Rickettsia prowazekii*, *Coxiella burnetii*, as well as viruses like human immunodeficiency virus (HIV) and simian immunodeficiency virus (SIV) (Neyrolles et al., 2006; Tanowitz et al., 2017). These findings highlight the potential role of adipose tissue as a niche for various microorganisms (Table 2). Moreover, Table 3 highlights the predominant microbial taxa and their functions in different health conditions, such as obesity, T2D, and obesity-associated T2D. However, the significance of the dormant state observed in certain microorganisms, allowing them to evade host defence mechanisms and drugs, in the context of obesity and related health conditions is currently unclear. In a recent report, it has been shown that in Crohn’s disease, gut bacteria translocated to mesenteric adipose tissue (Ha et al., 2020). This process contributes to the formation of “creeping fat” and obstructs the systemic translocation of gut bacteria. The expanded mesenteric adipose tissue in the sites of the gut barrier enriched with *C. innocuum* remodels the macrophage population toward the M2 phenotype, which helps prevent potentially harmful bacterial antigens that have translocated across the barrier from the gut lumen (Ha et al., 2020; Smith and Bénézech, 2020). These findings provide insights into the specific microbial compositions associated with different inflammatory conditions and their relationship with adipose tissue (Figure 1).

Overall, these studies shed light on the diverse microbial communities present in adipose tissue and their potential implications for obesity-induced inflammation and related health conditions. Further research is needed to fully understand the complex interactions between adipose tissue and the gutmicrobiota for their role in metabolic health and disease.

## 4 Gut-brain axis linked with obesity-induced type 2 diabetes

The gut-brain axis, commonly referred to as the feeding system, is a sophisticated feedback mechanism between the gut and the brain. It has long been known that the gut-brain axis is crucial for maintaining energy balance. The relationship of the gut microbiota with the enteric nervous system (ENS) and central nervous system (CNS) is beginning to be shown by more recent research.

### 4.1 Gut-brain axis in metabolic homeostasis

The gut-brain axis is a bidirectional network system of hormonal and neurological signalling cascades that are involved in neurologic, endocrine, immune, and metabolic pathways linking the ENS and CNS systems, and connecting the effect of gut microbiota on physiological health (Appleton, 2018). Signals from the gut in response to an influx of nutrients during a meal are traditionally conveyed to the brain, notifying the CNS about meal quantity and composition (Lam et al., 2009; Thorens, 2014; Fournel et al., 2017). The brain, especially the hypothalamus, integrates this information as well as other gut-derived signals to regulate the balance of dietary intake, consumption of energy, and glucose homeostasis (Peterhoff et al., 2003; Gautam et al., 2006). This postprandial gut feedback is mediated by entero-endocrine cells (EECs), which are specialized neuroendocrine cells of the intestinal epithelium. The EECs are found throughout the gut epithelium and respond to nutrient and mechanical stimuli by secreting hormones and neurotransmitters such as glucagon-like peptide 1 (GLP-1), gastric inhibitory polypeptide (GIP), cholecystokinin (CCK) (Gribble and Reimann, 2016), Peptide YY (Batterham et al., 2006), oxyntomodulin (OXM) (Schepp et al., 1996), ghrelin (Date et al., 2000), nesfatin (Stengel et al., 2009), serotonin (5-hydroxytryptamine), and Insulin-like peptide 5 (Insl5) (Grosse et al., 2014) which informs CNS particularly hypothalamus to coordinate and maintain metabolic homeostasis (Schwartz et al., 2000; Lam et al., 2009). These molecules critically influence the secretion of insulin, gastric acid, and bile acids, as well as gut motility and food intake via vagal afferent neurons of ENS or through the circulatory route (Ley et al., 2005; Cani et al., 2007; Duparc et al., 2011). Indeed, metabolites generated from the gut microbiota including SCFAs, butyrate, propionate, lactate, or mimetics such as γ-aminobutyric acid (GABA), and melanocyte-stimulating hormone (MSH)-mimetic, ClpB, can influence the release of these hormones and neurotransmitters (Newgard et al., 2009; Qin et al., 2012; Molinaro et al., 2020). Nutrient-induced gut peptides can function in a paracrine fashion by activating vagal neurons that innervate tissue surrounding the intestinal epithelium and signal to the brain, or in an endocrine fashion by targeting the brain and other peripheral organs involved in metabolic regulation (Köhler et al., 2003). Under normal conditions, intestinal glucose sensors, like SGLT1, and TASR1/2 initiate a signal to the afferent neurons for generating nitric oxide (NO) in the hypothalamus to allow glucose entry into the tissue by stimulating the autonomic nervous system (ANS) (Fournel et al., 2017). The intervention of ANS in pancreatic islets’ parasympathetic and sympathetic nerves regulates the islet hormone secretion (Peterhoff et al., 2003; Gautam et al., 2006; Thorens, 2014).

### 4.2 Gut microbial metabolites modulate gut-brain axis in type 2 diabetes

Gut microbiota maintains glucose homeostasis by directly communicating with the brain via microbe-derived metabolites such as SCFAs. The SCFAs activate G-protein coupled receptors (GPRs), FFAR2 (free fatty acid receptor 2), and FFAR3 (free fatty acid receptor 3) localized in EECs (Kasubuchi et al., 2015), resulting in gut peptide release. In healthy intestinal microbiota, the majority (approximately 90%) of known phylogenetic categories consist of Bacteroidetes and Firmicutes, including genera such as *Ruminococcus*, *Lactobacillus*, *Clostridium*, and to a lesser extent *Actinobacteria*, *Verrucomicrobia*, and *Fusobacteria* (Eckburg et al., 2005). These bacteria play important roles in maintaining intestinal health and normal physiological functions. However, in individuals with T2D, there are notable changes in the composition of the intestinal microbiota that includes opportunistic pathogenic bacteria such as *Bacteroides caccae*, *Clostridium hathewayi*, *Clostridium symbiosum*, *Eggerthella lenta*, *Clostridium ramosum*, and *Escherichia coli* are found to be more abundant (Qin et al., 2012). Additionally, mucin-degrading bacteria like *Akkermansia muciniphila*, sulfate-reducing bacteria like *Desulfovibrio* Sp., and imidazole propionate-producing bacteria such as *Clostridium baumannii*, *Clostridium parasymbiotics*, and *Ruminococcus gnavus* have also been linked to T2D (Molinaro et al., 2020). On the other hand, butyrate-producing bacteria such as *Clostridiales*, *Faecalibacterium prausnitzii*, *Roseburia intestinalis*, *E. rectale*, and *Roseburia inulinivorans*, which are important for gut health and energy metabolism, are significantly decreased in individuals with T2D (Qin et al., 2012). In the case of db/db mice, a commonly used mouse model for studying T2D pathophysiology, it has been observed that these mice experience intestinal inflammation, which can disrupt glucose metabolism (Duparc et al., 2011). This inflammation may be induced by an increase in NO production which impacted the disturbances in glucose metabolism contributing to the progression and severity of T2D in these mice. In addition, prebiotic-induced improvements in glucose homeostasis are contingent on GLP-1R signaling implying that prebiotic-induced microbiome changes repair the gut-brain axis (Jin et al., 2013). Increasing peptide production in prebiotic therapy enhances gut barrier integrity in the context of high-fat eating and obesity, which ultimately lowers the circulatory levels of LPS (Thorens, 2011). Recent research also identified small intestinal microbiota as a key mediator of the gut-brain axis in glucose homeostasis. Direct small intestinal infusion of *Lactobacillus gasseri* rescues intestinal lipid sensing, which is consistent with changes in the small intestinal microbiota regulating nutrient-induced gut-brain transmission (Biessels and Reagan, 2015; Omori et al., 2022). *Lactobacillus gasseri* expresses bile salt hydrolase and improvements in lipid sensing were dependent on the decreased farnesoid-X receptor (FXR) signalling, emphasizing the role of bile acids in gut-brain signalling processes that regulate metabolic homeostasis (Omori et al., 2022). Changes in the proportions of Gram-positive compared to Gram-negative bacteria in the intestinal lumen have a major impact on LPS bioavailability (Qin et al., 2012). The HFD-induced leaky gut helps to form an interaction of the bacterium with the intestinal epithelial cells by three successive pathways: a) attachment and invasion, b) alteration in the epithelial barrier, and c) inducing inflammation (Köhler et al., 2003). This allows tissue microbe-associated molecular patterns (MAMPs) and LPS to translocate from the intestinal lumen to the circulatory system (Delzenne and Cani, 2011). A crucial defence against gut-derived bacterial products getting into the bloodstream is provided by intestinal inflammatory cells (Steenbergen et al., 2015). It was discovered that microbiota-driven LPS, through its interactions with myeloid differentiation factor 2 (MD-2), TLR4, and CD14 contributes to the low-grade tissue inflammation in metabolic diseases associated with endotoxemia (Delzenne and Cani, 2011; Steenbergen et al., 2015). Similarly, microbiota-emanated peptidoglycans upon binding with NOD2 can modulate the intestinal inflammatory milieu thus influencing glucose tolerance and insulin sensitivity (Cani et al., 2008).

Upon activation of the pro-inflammatory cytokines by intestinal innate and adaptive immune cells, the activities of intrinsic and extrinsic enteric sensory neurons are altered (Kindt et al., 2010; Buckley et al., 2014). Intestinal cells send an abnormal nerve message that does not increase hypothalamic NO release and leads to dysfunctionality in ENS neurons (Gonzalez-Correa et al., 2017). The elevated level of TNF-α negatively impacts gut motility by upregulating the expression of cyclooxygenase 2 (COX-2) (Rehn et al., 2005) and enteric neuron apoptosis (Chandrasekharan et al., 2013). Additionally, IL-1β accelerates the diarrhoea phase by depolarizing the membrane potential, and decreasing the membrane conductance in obese/diabetic patients (Acosta and Camilleri, 2014). It also reported that a high accumulation of inflammatory markers induces the expression of serotonin and GABA, inhibiting brain functionality (Wang et al., 2012; Jin et al., 2013). Dysfunctionality in the gut-brain axis is driven by the interruption of the brain-islet axis and leads to islet abnormalities (Thorens, 2011), which leads to the failure of the insulin-regulated cognition and neuronal plasticity in the CNS (Biessels and Reagan, 2015). Moreover, it has been discovered that *E. coli* generates ClpB which regulates food intake (Breton et al., 2016). GF mice that lack any microbiome exhibit lower adiposity and higher insulin sensitivity despite greater food intake (Chandrasekharan et al., 2013), and they are protected against diet-induced obesity (Acosta and Camilleri, 2014). In comparison to ordinary mice, GF mice have altered gut-brain metabolic communication with alterations in EEC quantity as well as differences in intestinal nutrient-sensing machinery and nutrient-induced gut peptide release (Wang et al., 2012). Thus, ENS signalling deficiencies in GF mice lead to changes in the gut-brain axis that modulates energy balance exhibiting diminished activation of brainstem neurons.

Overall, these findings highlight the importance of a balanced and diverse intestinal microbiota in maintaining metabolic health. Alterations in the composition of the microbiota, characterized by changes in specific bacterial genera, can contribute to the development and progression of T2D and associated metabolic dysfunctions.

## 5 Gut microbiome-derived metabolites promote pancreatic β-cell dysfunction in obesity

Obesity-associated metabolic disturbances can impair the function of the pancreas, particularly in insulin-secreting β-cells and it has been unequivocally proven in prior studies that β-cell mass reduces before the development of T2D (Weir et al., 2020). The molecular reasons for β-cell depletion were thoroughly discussed in several research articles and reviewed elsewhere (Kahn, 2001; Prentki and Nolan, 2006; Cerf, 2013; Swisa et al., 2017). Although none of the direct evidence showed an existing pancreatic microbiome, there are a plethora of reports that prove gut microbiome-derived metabolites are responsible for β-cell dysfunction and insulin resistance. Healthy gut microbiota supports pancreatic cell expansion and maintenance of cell growth, as such early zebrafish pancreatic cell growth required certain gut microbiota such as some *Aeromonas* strains that can release cell expansion factor A (BefA), which encouraged proliferation and thereby cell growth (Hill et al., 2016; Zhou et al., 2022). More crucially, the specific bacterial species in humans could also release proteins that functioned similarly to BefA-like proteins leading to the development of novel T2D therapy strategies (Hill et al., 2016). Obesity exerts a multifaceted impact on the pancreas, as well as the composition and function of the gut microbiome. These alterations contribute to metabolic disturbances and insulin resistance, playing a significant role in the development and progression of obesity-related metabolic disorders. The β-cell death in T2D has been linked to many variables, including hyperglycemia, amyloid build-up, oxidative or endoplasmic reticulum stress, inflammatory cytokines, dysfunctional autophagy, and lipotoxicity (Lv et al., 2022).

### 5.1 Metabolites of gut microbiome affect β-cell function and insulin sensitivity

BCAAs such as leucine, isoleucine, and valine serve as important signalling molecules in the body, exerting both direct and indirect effects on the insulin signalling pathway. While BCAAs have shown potential anti-obesity effects in rodent models, it is noteworthy that individuals with obesity often exhibit elevated levels of circulating BCAAs. One proposed mechanism linking elevated BCAA levels and T2D involves the activation of the mTORC1by leucine, one of the BCAAs. Activation of mTORC1 by leucine can lead to the uncoupling of insulin signalling at an early stage, potentially contributing to insulin resistance. On the contrary, the activation of mTORC1 in β-cells has been associated with a protective effect against the development of T2D. This activation is linked to compensatory increases in islet and β-cell mass, which help to prevent the onset of T2D. An alternative model, known as the BCAA dysmetabolism model, suggests that it is not the BCAAs themselves but the accumulation of metabolites derived from BCAA metabolism that contributes to β-cell mitochondrial dysfunction, stress signalling with mitotoxic effects, and apoptosis associated with T2D (Lynch and Adams, 2014).

L-tryptophan (Trp), an exogenous essential amino acid acts as a metabolic precursor, for melatonin (Leja-Szpak et al., 2004) and serotonin (Almaça et al., 2016). Melatonin rescues the pancreatic cells against acute pancreatic damage by oxidative stress (Leja-Szpak et al., 2004). Almaca et al. have revealed that serotonin acts as a paracrine signal released by pancreatic β-cells to regulate glucagon secretion. The study demonstrated that without serotonin signalling, α-cells fail to respond appropriately to glucose level alteration (Almaça et al., 2016). Tryptophan metabolism involves three main pathways: the kynurenine pathway through indoleamine 2,3-dioxygenase 1 (IDO1), the serotonin production pathway via Trp hydroxylase 1 (TpH1), and the direct transformation of Trp into various molecules by the gut microbiota, including ligands of the aryl hydrocarbon receptor (AhR). The kynurenine pathway is the predominant route of tryptophan breakdown in most mammalian cells. Within this pathway, kynurenines can inhibit proinsulin synthesis in pancreatic islets and form complexes with insulin, reducing its biological activity and promoting the development of insulin resistance (Song et al., 2017; Vangipurapu et al., 2020).

The gut microbiome produces various metabolites, such as SCFAs and bile acids which can influence pancreatic function and insulin secretion (Kaneto et al., 2022). Pancreatitis is associated with alteration in the composition of the gut microbiota, particularly at the phylum level, characterized by an increase in Proteobacteria and a decrease in strains that produce SCFAs. A study by Yu et al. revealed that *Eubacterium hallii*, a prominent bacterium responsible for producing butyrate, was significantly depleted in pancreatitis patients and the loss of butyrate-producing bacteria is attributed to increased oxidative stress in the pancreas (Yu et al., 2020). Moreover, butyrate has been shown to ameliorate pancreatitis by suppressing the activation of NF-κB and decreasing HMGB1 expression (Pan et al., 2019). The SCFAs such as propionate have been associated with insulin secretion and sensitivity. It has been shown that SCFAs stimulate the production of GLP-1 through the activation of FFAR2, also known as GPR43 (G-protein coupled receptor 43), regulate glucose-dependent insulin secretion from pancreatic β-cells, and inhibit glucagon secretion. This mechanism contributes to the maintenance of glucose homeostasis (Silva et al., 2020).

The gut-derived metabolite trimethylamine N-oxide (TMAO) has been linked to obesity, and elevated levels of TMAO have been found to positively correlate with the presence of T2D. Krueger et al. showed the effects of TMAO on insulin secretion by β cells were investigated, revealing that TMAO exposure did not contribute to the development of T2D but instead exhibited beneficial effects (Krueger et al., 2021). Interestingly, TMAO exposure demonstrated a protective effect against glucolipotoxicity (GLT)-induced damage to β-cells by reducing oxidative stress and preserving insulin granule formation, which suggests that TMAO promotes the preservation of functional β-cell mass, thus counteracting T2D-promoting mechanisms (Krueger et al., 2021).

### 5.2 Oxidative stress and inflammation in pancreatic β-cell associated with microbial components

Chronic hyperglycemia, a characteristic feature of T2D, is linked to the rapid formation of advanced glycation end products (AGEs) and glucose autoxidation (Robertson et al., 2003). These processes further lead to the production of reactive oxygen species (ROS) and oxidative stress. In β-cells, superoxide (O2-), hydroxyl (OH) radicals, hydrogen peroxide (H2O2), and other ROS molecules are among the many that are being intensively investigated for their harmful impact in exacerbating diabetes-related problems (Kulkarni et al., 2023). Reactive oxygen species (ROS) and reactive nitrogen species (RNS) overproduction or improper clearance lead to oxidative stress, which may have a variety of harmful consequences on cellular metabolism (Hasnain et al., 2016). ER stress during protein misfolding results in unfolded protein response (UPR) may impair insulin transcription and translation and trigger inflammation and death. The SAPKs, p38, and JNK are examples of stress-sensing pathways that are activated by oxidative stress and cause damage to cells (Houet al., 2008). Reports have indicated that malfunctioning pancreatic islets exhibit relatively low levels of antioxidant enzymes such as copper/zinc superoxide dismutase (Cu/Zn-SOD), manganese superoxide dismutase (Mn-SOD), catalase, and glutathione peroxidase (GPx) (Robertson et al., 2003; Stancill et al., 2019). As a result, they become more vulnerable to oxidative damage, eventually resulting in the death of β-cells and it is clearly showing that there is a clear connection between oxidative stress, mitochondrial dysfunction, and T2D (Rocha et al., 2020).

**FIGURE 3:**
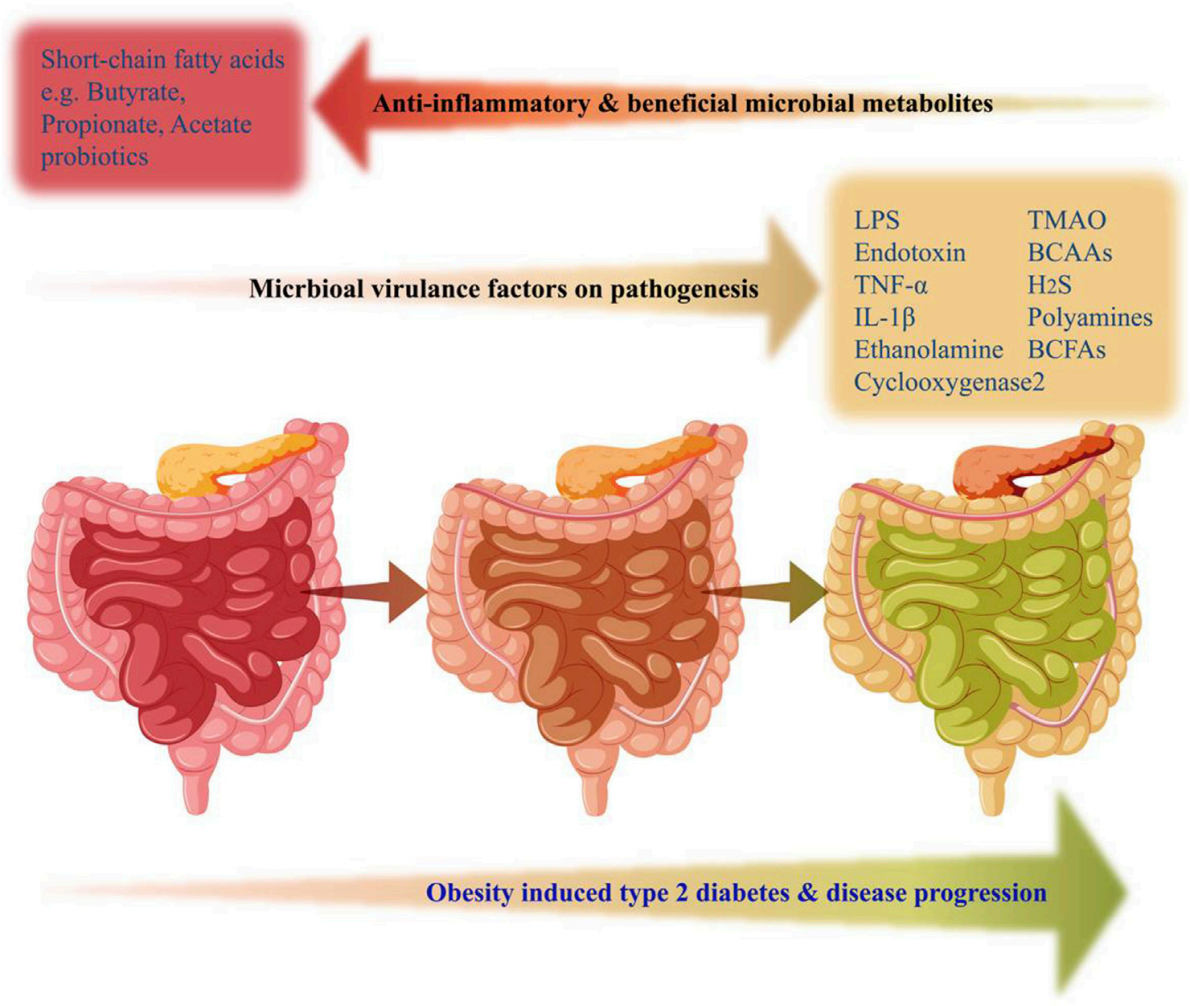
Gut microbial-derived factors alteration with the progression of obesity and type 2 diabetes. Increased production of several metabolites associated with gut dysbiosis such as LPS, TNFα, TMAO, H2S, BCAAs, BCFAs, and Polyamines were linked with the progression of obesity-induced T2D. On the contrary, beneficial microbiome-derived metabolites from the healthy gut, responsible for anti-inflammatory effects and physiological homeostasis, such as Propionate, Acetate, and Butyrate (short-chain fatty acids) were reduced in obesity-induced gut dysbiosis. The gut represented in pink color indicates a healthy state, whereas, brown color and green color indicate obesity-induced pathophysiological progression to T2D state.

Obesity and T2D also feature higher release of free fatty acids (FFAs), which modulatesignalling pathways related to glucose metabolism, and β-cell function. Elevated FFAs inhibit glucose-stimulated insulin secretion and lead to β-cell dysfunction through cytotoxic mechanisms, including apoptosis. FFAs exposure is associated with ceramide synthesis, mitochondrial dysfunction, overexpression of apoptotic genes, and intracellular triglyceride accumulation in β-cells mediated by sterol regulatory element-binding proteins (SREBPs) (Martínez-Montoro et al., 2022). Visceral adipose tissue releases pro-inflammatory factors like IL-2, IL-6, IL-8, IL-12A, and MCP-1 in obese conditions, which can contribute to β-cell dysfunction. Macrophages present in adipose tissue are the early responders and play a crucial role in promoting the pro-inflammatory environment that negatively impacts pancreatic β-cells (Daniele et al., 2014). Vangipurapu et al. reported an increase in sphingolipids (e.g., myoinositol) and fatty acids from early analysis of pathway enrichment and plasma metabolite analysis of chronic pancreatitis patient samples (Vangipurapu et al., 2020). Gao et al. revealed that the accumulation of microbial DNAs contributes to inflammation and abnormalities in pancreatic islet β-cells through their observations of passing the extracellular vesicles containing microbial DNA from the gut to β-cells (Gao et al., 2022). Bacterial LPS trigger inflammatory responses and impact pancreatic β-cell function (Wu et al., 2023). These metabolites can directly affect pancreatic function and also influence systemic inflammation, gut barrier integrity, and other metabolic pathways. Individual variations in gut microbial composition and metabolism further contribute to the complexity of this relationship (Figure 3). Further research is needed to fully understand the mechanisms by which microbial metabolites contribute to pancreas dysfunctions in T2D and explore potential therapeutic targets.

## 6 Therapeutic approaches to manage type 2 diabetes by targeting gut microbiota

Therapeutics classes including probiotics, prebiotics, FMT, and microbial-derived molecules, offer potential strategies for controlling insulin secretion and insulin resistance (Zhang et al., 2019). Probiotics, such as *Lactobacillus* spp. and *Bifidobacterium* spp., have shown promise in improving glycemic control and insulin sensitivity (Salles et al., 2020). Prebiotics, as dietary fibers, can selectively promote the growth of beneficial gut bacteria and improve glucose metabolism (Holscher, 2017). Moreover, FMT is being explored for T2D management. Microbial-derived molecules like SCFAs have implications for metabolic processes. However, further research is needed to optimize these approaches and personalize interventions for effective microbiome-based therapies in T2D. Probiotics, live microorganisms that confer health benefits to the host, have been shown to improve insulin sensitivity and glucose tolerance in both animal and human studies. Several studies have reported that probiotics can lead to improvements in markers of inflammation, glycemic control, and blood pressure. Probiotic supplementation has been shown to reduce c-reactive protein (CRP) levels, indicating a potential anti-inflammatory effect, along with the improvement of HbA1c, fasting plasma glucose, and fasting insulin levels (Wang et al., 2021). Consumption of probiotics has been associated with a decrease in serum cholesterol levels and reduced cholesterol absorption in the intestines which inhibits the activity of the HMG-CoA reductase, an enzyme critically involved in endogenous cholesterol synthesis (Kumar et al., 2012).

The biological effects of *Lactobacillus* spp. and *Bifidobacterium* spp., on glucose intolerance and insulin resistance were majorly studied in animal models of T2D. *Lactobacillus plantarum* CCFM0236 has shown beneficial effects in improving insulin resistance, reducing systemic inflammation, and ameliorating pancreatic β-cell dysfunction in HFD-induced T2D animal models. This probiotic strain reduced insulin resistance and preserved pancreatic β-cell function, leading to better glycemic control (Li et al., 2016). Clinical trial studies revealed *Lactobacillus reuteri* DSM 17938, *Lactobacillus case* 431, *Lactobacillus acidophilus*, *Bifidobacterium lactis*, *Bifidobacterium bifidum*, *Lactobacillus salivarius* W24 improves insulin sensitivity index, reduces HbA1c and HOMA-IR. Moreover, strains of *Lactobacillus*, *Lactococcus*, *Bifidobacterium*, *Propionibacterium,* and *Acetobacter* genera supplementation significantly reduced pro-inflammatory cytokines including TNF-α, IL-1β and improved physiological glycemic control by lowering HOMA-IR and HbA1c (Zhai et al., 2021).

Combined supplementations of probiotics and prebiotics have also been shown to improve glucose metabolism and reduce insulin resistance. Prebiotics normalizes the GI tract’s pH value, reduce hyperlipidemia, and improve the absorption of cation ions, creating an environment favourable for the growth of beneficial bacteria (Megur et al., 2022). In addition, FMT has shown promise in improving glycemic control in patients with T2D. The recipients of FMT showed a significant improvement in insulin sensitivity, indicating a positive effect on glucose metabolism. This finding was further supported by a larger-scale follow-up study where the recipients exhibited a reduction in HbA1c levels at 6 weeks post-intervention (Kootte et al., 2017). However, the insulin sensitivity of the recipients and the composition of their gut microbiota returned to baseline after 18 weeks post-intervention (Kootte et al., 2017). This suggests that the effects of FMT on insulin sensitivity and gut microbiota composition may be transient and not sustained in the long term.

## 7 Summary

The altered gut microbial community leads to disruptions in metabolic homeostasis causally linked with the imbalance of lipid homeostasis and chronic inflammation which is associated with obesity and T2D. Remodelling of microbial profiles and microbiome-derived metabolites contributes to the development of insulin resistance by modulating the gut-brain axis, loss of pancreatic functionality, and white adipose tissue dysfunction. Additionally, specific microbial genera were found in the visceral adipose tissue that influences its normal function during obesity. Moreover, pathogenic microbes such as *Escherichia* and *Shigella* were abundantly present in obese diabetic patients. The applications of prebiotics, probiotics, or faecal microbial transplantation are considered contemporary approaches that can be offered to manage T2D. Here we consolidate the current knowledge in this field and underscore the urgent need for further research and clinical interventions to combat the rising epidemic of obesity-induced metabolic disorders.

## 8 Future perspective

In recent years, the study of gut and adipose tissue dysbiosis in obese individuals with T2D has attracted significant attention, and researchers are exploring several therapeutic approaches in this field. One potential way is the development of personalized microbiome-based therapies. Another important aspect is gaining a mechanistic understanding of how gut dysbiosis contributes to obesity and T2D for developing targeted therapeutics. It is needed to understand the potential implications and mechanisms underlying the relationship between microbial dormancy and obesity. This review raises a few fundamental questions to understand the basics of obesity-induced pathogenesis. Such as, how microbial compartmentalization occurs within the visceral adipose tissue (VAT) during obesity and how the obese adipose tissue microenvironment supports microbial survival. It also demands the ongoing search for the source and origin of tissue-specific microbes. Moreover, what are the factors that govern gut and adipose tissue dysbiosis in obesity and actively control microbial localization in insulin-responsive organs such as the liver and adipose tissue? Therefore, further research is needed to fully comprehend the gut-adipose tissue axis in obesity and its role in the progression of insulin resistance and T2D, as our understanding of this subject is still in its nascent stages.
